# Meningococcal pneumonia: a review

**DOI:** 10.1186/s41479-019-0062-0

**Published:** 2019-08-25

**Authors:** Charles Feldman, Ronald Anderson

**Affiliations:** 1 Department of Internal Medicine, Faculty of Health Sciences, University of the Witwatersrand, Johannesburg, South Africa; 2 Department of Immunology and Institute for Cellular and Molecular Medicine, Faculty of Health Sciences, University of Pretoria, Pretoria, South Africa

**Keywords:** Antibiotics, Chemoprophylaxis, Diagnosis, *Neisseria meningitidis*, Pathogenesis, Pneumonia, Risk factors, Vaccination

## Abstract

**Background:**

Although *Neisseria meningitidis* is one of the major causes of meningitis, meningococcal pneumonia is the most common non-neurological organ disease caused by this pathogen.

**Methods:**

We conducted a review of the literature to describe the risk factors, pathogenesis, clinical features, diagnosis, treatment and prevention of meningococcal pneumonia.

**Results:**

Meningococcal pneumonia was first described in 1907 and during the 1918–1919 influenza pandemic large numbers of cases of meningococcal pneumonia occurred in patients following the initial viral infection. A number of publications, mainly case series or case reports, has subsequently appeared in the literature. Meningococcal pneumonia occurs mainly with serogroups Y, W-135 and B. Risk factors for meningococcal pneumonia have not been well characterised, but appear to include older age, smoking, people living in close contact (e.g. military recruits and students at university), preceding viral and bacterial infections, haematological malignancies, chronic respiratory conditions and various other non-communicable and primary and secondary immunodeficiency diseases. Primary meningococcal pneumonia occurs in 5–10% of patients with meningococcal infection and is indistinguishable clinically from pneumonia caused by other common pathogens. Fever, chills and pleuritic chest pain are the most common symptoms, occurring in > 50% of cases. Productive sputum and dyspnoea are less common. Diagnosis of meningococcal pneumonia may be made by the isolation of the organism in sputum, blood, or normally sterile site cultures, but is likely to underestimate the frequency of meningococcal pneumonia. If validated, PCR-based techniques may be of value for diagnosis in the future. While penicillin was the treatment of choice for meningococcal infection, including pneumonia, prior to 1991, a third generation cephalosporin has been more commonly used thereafter, because of concerns of penicillin resistance. Chemoprophylaxis, using one of a number of antibiotics, has been recommended for close contacts of patients with meningococcal meningitis, and similar benefits may be seen in contacts of patients with meningococcal pneumonia. Effective vaccines are available for the prevention of infection with certain meningococcal serogroups, but this field is still evolving.

**Conclusion:**

Meningococcal pneumonia occurs fairly frequently and should be considered as a possible cause of pneumonia, particularly in patients with specific risk factors.

## Introduction

Pneumonia due to *Neisseria meningitidis* (meningococcus) was first reported by Jacobitz in 1907, in patients with pneumonia in whom *N. meningitidis* was isolated from sputum samples [1]. The initial cases were soldiers sharing barracks, who were probably infected through a single index patient [2]. Interestingly, even all those years ago, Jacobitz had recognised that healthy individuals could harbour *N. meningitidis* in their oropharynges such that isolation of the microorganism could not provide a definitive clinical diagnosis of meningococcal pneumonia [1, 2]. During the 1918–1919 influenza pandemic a large number of cases of meningococcal pneumonia were described in patients following on the initial viral infection [3]. Since the description of these initial cases, several surveillance studies, case series, as well as a myriad of case reports have described cases of meningococcal pneumonia, occurring predominantly in adolescents, adults and the elderly [2]. Most recently, Vossen and colleagues reviewing the literature were able to identify 344 cases reported in the Americas, Europe, Australia and Asia between 1906 and 2015 [2]. These authors also noted that when reviewing national surveys on the incidence of invasive meningococcal disease (IMD), pneumonia was noted to be the most common non-neurological organ disease, occurring in 66 of 364 cases (17% of cases). In this context, pneumonia associated with meningococcal disease may be primary, when it occurs alone without evidence of pre-existing meningitis or meningococcaemia [4], but can also occur in association with, or as a complication of, both those conditions and/or with other manifestations of meningococcal infection. The meningococcal pneumonia cases published in the literature include both primary and/or associated pneumonias. However, not all authors describe the cases separately, such that distinguishing true primary infections in the literature is often difficult.

### Neisseria meningitidis

#### The microorganism

*N. meningitidis* (meningococcus) is a Gram-negative aerobic diplococcus. It has a typical bean or kidney shape, and is an obligate human pathogen [5–7]. The microorganism frequently colonises the oro- or nasopharynges of even healthy individuals, but can also colonise other parts of the body [7]. The microorganism grows well on chocolate and blood agar at temperatures between 35 and 37 °C in an atmosphere of 5–10% carbon dioxide [8, 9], while confirmation of the presence of this microorganism in clinical specimens is undertaken according to the results of a series of carbohydrate fermentations [8]. The microorganism has a number of virulence factors (see below), including a polysaccharide capsule. While this capsule is important for protecting the microorganisms from complement-mediated phagocytosis and lysis, it also enables differentiation of the 13 serogroups of the microorganism, of which, serogroups A, B, C, W, X and Y are largely responsible for human disease [5–7].

#### Epidemiology of meningococcal infection

Although meningococcal disease is widespread globally, the epidemiology of this condition varies in different geographical regions and has evolved and changed over time [10–14]. The disease can occur as sporadic cases, outbreaks, or as large epidemics [11]. Epidemics with large numbers of infected patients occur intermittently in the sub-Saharan African region (often referred to as the “meningitis belt”), with periodicity of five to 10 years [5–7]. In countries such as Brazil, however, it tends to be endemic, with periodic occurrence of epidemics [7]. In the developed world, the infection tends to be endemic and somewhat restricted [5–7], although in countries such as the United States (US), there has been an increase in outbreaks, as well as a change in the serogroups commonly causing infection [5, 14].

The distribution of the different serogroups varies both temporally and geographically, which may explain the varied clinical presentations of the infection regionally and chronologically [7, 11]. Importantly, knowledge of the distribution of serotypes in different regions is essential for the design of vaccination programs [5]. Seasonally, infections are more common in winter, or early spring. Endemic infections occur mostly in children below the age of 10 years, while during epidemic infections a wide age range of patients is infected [7]. In general, the infection is more common in men, but in patients older than 50 years, it is more common in women [7]. With respect to transmission, the development of IMD is related to the recent acquisition of a pathogenic strain by a susceptible individual, most commonly as result of close physical contact [8].

### Risk factors for meningococcal infection

#### Patient risk factors

Age is an important risk factor for meningococcal pneumonia, which occurs mostly in older individuals > 50 years of age, and is the most common manifestation of IMD in those aged > 65 years, in contrast to meningococcal meningitis, which occurs predominantly in children and adolescents [2, 15]. However, more recent data suggests that the age distribution of meningococcal pneumonia is bimodal, occurring before the age of 30 years and after 60 years of age [2]. Although all serogroups of the meningococcus can cause pneumonia, the less common serogroups of the pathogen are more frequently implicated as discussed below [2, 8].

Other risk factors for IMD include smoking, immunoglobulin and complement deficiencies, asplenia, deficiencies in mannose-binding lectin and other genetic abnormalities [2, 16, 17] and possibly human immunodeficiency virus (HIV) infection [15, 18, 19]. Additional risk factors include close contact with patients who have meningococcal infection, people living in close quarters (such as military recruits [as mentioned above], university students, and people on Hajj), as well as preceding influenza and other respiratory viral and bacterial infections including those caused by *Haemophilus influenzae* and *Streptococcus pneumoniae* [16, 17, 19, 20]. Winstaed in his series of cases with meningococcal pneumonia, collected over a 25 year period, noted concomitant viral infection, as well as underlying lymphoma, multiple myeloma, asthma, chronic obstructive pulmonary disease (COPD), insulin-dependent diabetes mellitus and other forms of diabetes mellitus, systemic lupus erythematosus, HIV infection, sickle cell anaemia, coronary artery disease/patients with coronary artery bypass graft, and cirrhosis among his patients [19]. These risk factors are summarised in Table 1.

**Table 1:** Possible risk factors for invasive meningococcal disease and/or meningococcal pneumonia

• Age (older individuals)	
• Smoking	
• Close contact with persons with meningococcal infection	
• People living in close quarters (e.g. military recruits, university students, Hajj)	
• Chronic respiratory conditions (asthma, COPD)	
• Coronary artery disease (or CABG)	
• Diabetes mellitus	
• Cirrhosis	
• HIV infection	
• Systemic lupus erythematosus	
• Sickle cell anaemia (or asplenia)	
• Deficiencies in mannose-binding lectin and other genetic abnormalities	
• Preceding viral infection (especially influenza)	
• Preceding bacterial infection (including *S. pneumoniae* and *H. influenzae*)	
• Meningococcal serogroups Y, W-135, B	
• Immunoglobulin and complement deficiencies	
• Haematological malignancies (lymphoma, myeloma)	

*CABG* coronary artery bypass graft, *COPD* chronic obstructive pulmonary disease, *HIV* human immunodeficiency virus

#### Specific meningococcal serogroups

Studies have suggested that the frequency of serogroup Y, which was previously relatively uncommon in Europe, remains high or is increasing in certain regions of the continent [21], with a number of studies and case reports having indicated the association of serogroup Y with meningococcal pneumonia [22–26]. In England and Wales, increasing numbers of laboratory-confirmed cases of IMD caused by serogroup Y were detected between 2007 and 2009, with clonal type cc174 found to be associated with non-meningeal disease, particularly pneumonia, in the elderly (> 65 years) [22]. Among those infected with serogroup Y, the median age of onset was highest for patients with pneumonia (86.1 years [IQR 69.9–90.0] versus 42.8 [IQR 14.4–71.7]; *p* < 0.0001). Persons with pneumonia were more likely to have underlying comorbid conditions (13/19 [68%] versus 12/46 [26%]; *p* = 0.001), while the case fatality rate was substantially higher for pneumonia than meningitis (9/19 [47%] versus 2/22 [9%]), septicaemia (1/17 [6%]) or other conditions (no deaths). After adjusting for age, underlying medical conditions and pneumonia (OR 7.0; 95%CI 1.4–36.4; *p* = 0.020) were found to be independently associated with death. Infections caused by serogroup Y also increased significantly in Sweden between 1995 and 2012, with one retrospective observational study of 175 patients documenting the occurrence of pneumonia in 34 (19%) patients, either alone or associated with another focus of infection [26]. In that study, the highest mortality was for cases with bacteraemia with no known focus (13%), while the mortality for those cases with pneumonia was 9%.

A number of studies and case reports from the US has also attested to the importance of serogroup Y as a cause of meningococcal pneumonia, either on its own, or associated with other forms of meningococcal infection [27–29]. Additional studies in the military from the US [30–32] and Finland [33] have also documented serogroup Y as a cause of meningococcal pneumonia. In one series of 12 cases of primary meningococcal pneumonia reported in the US, serogroup Y infection was documented in two cases, confirmed by radiological evidence of a pneumonia and isolation of the organism on blood cultures [30]. In another such study, serogroup Y meningococcal disease occurred in 88 US Air Force recruits, of whom 68 had primary bacterial pneumonia, this being much more common than the occurrence of meningococcaemia or meningitis [31]. All cases ultimately responded well to antibiotic therapy. In the Finnish study, a fulminant case of meningococcal pneumonia complicated by sepsis, pericarditis and pleural effusion prompted the investigation of the distribution of meningococcal serogroups in nasopharyngeal cultures among the recruits from the same unit [33]. The study documented that 14 of 31 (46%) isolates cultured were serogroup Y, isolated almost entirely from individuals that had been vaccinated against serogroups A and C.

Similarly, a number of studies, mainly case reports or small case series, have also noted that serogroup W-135 is associated with pneumonia, particularly in older patients [34–41]. The latter study, a nationwide retrospective study, which analysed epidemiological data from 115 patients with laboratory-confirmed meningococcal disease found that serogroup W-135 accounted for 26% of all cases, and that most of the patients (76.7%) were older than 20 years of age [41]. No differences were found in the presenting features other than a higher prevalence of pneumonia in those with W-135 infection (23.8% versus 1.5%; OR 20.6; 95% CI 2.3–189.0; *p* = 0.003) [41]. Lastly, a case report from the US described a 16-year old student with clinical signs and radiological findings compatible with pneumonia, which on sputum culture revealed a serogroup B meningococcus [42].

### Pathogenesis

#### Colonisation of the oro-nasopharynx by Neisseria meningitidis

As stated by Laver et al., *Neisseria meningitidis* “is one of a handful of potential pathogens that can silently colonise the oro-nasopharynx, which represents its sole biological niche” [43]. The estimated frequencies of oro-nasopharyngeal colonisation by this exclusively human pathogen are around 10–35% and 5.9% of adults and children, respectively [44]. As mentioned above, colonisation rates are significantly increased by both active cigarette smoking and exposure to sidestream tobacco smoke [45–47], probably as a consequence of smoke-mediated suppression of the innate and adaptive immune mechanisms of the respiratory tract [48, 49]. Cigarette smoke also affects respiratory pathogens directly, resulting in augmentation of bacterial virulence mechanisms, particularly increased biofilm formation [50–52].

The most important virulence factors of the meningococcus with respect to oro-nasopharyngeal colonisation include the immunoglobulin A1 (IgA1) protease and the anti-phagocytic polysaccharide capsule, as well as a series of epithelium-binding bacterial adhesins. The IgA1 protease, which is most important in previously exposed individuals, is a site-specific serine protease. It cleaves the antibody molecule at the hinge region, separating the Fab and Fc regions, preventing expulsion of the pathogen by mucociliary clearance, favouring attachment to respiratory epithelium [53]. It has also been proposed that the meningococcal IgA1 protease may have a broader than previously realised substrate specificity, encompassing other immune-associated proteins such as the type II tumour necrosis factor receptor [54] and human lysosome-associated membrane protein [55].

Notwithstanding its primary role in counteracting phagocytosis, the polysaccharide capsule of the meningococcus repels mucus, also hindering mucociliary clearance [56]. The capsular polysaccharides of four of the six major serogroups associated with invasive disease, specifically serogroups B, C, W, and Y are composed of sialic acid derivatives. The capsules of the other invasive groups, serogroups A and X, are composed of repeating units of O-acetylated (α1 → 6)-linked N-acetyl-D-mannosamine-1-phosphate [57] and (α-1 → 4) *N*-acetylglucosamine 1-phosphate [58], respectively. Sialic acids are also present in the cell surface glycoconjugates of eukaryotic cells and protect not only host cells, but also those meningococcal serogroups with sialic acid –containing capsules, against attack by the alternative pathway of complement via binding of the major regulatory protein factor H (fH), which is achieved via inhibition of C3 convertase and inactivation of C3b [59].

The next step in colonisation of the nasopharynx by the meningococcus involves attachment of the pathogen to respiratory epithelium, a process that necessitates a reduction in capsule size in order to expose underlying protein adhesins. Initial contact between the pathogen and epithelium is mediated by Type IV pili; these are complex structures comprised of over twenty proteins, which contribute to the formation of the fully functional adhesin, with the PilE and PilV components playing prominent roles in adhesion [60, 61]. The identity of the epithelial cell receptor targeted by the Type IV pilus has, however, remained elusive. A possible contender identified in brain endothelial cells is CD147 (also known as EMMPRIN or extracellular matrix metalloproteinase inducer) [62]. Although also expressed on epithelial cells, the involvement of CD147 in Type IV pilus-mediated attachment of the meningococcus to respiratory epithelium remains uncertain.

Type IV pilus-mediated epithelial attachment enables subsequent firmer binding mediated by the meningococcal opacity proteins, Opa and Opc [60]. Opa targets the carcinoembryonic antigen cell adhesion molecule (CEACAM) receptor on epithelium, mostly the CEACAM1 receptor [63], while Opc has been reported to bind to activated vitronectin on endothelial cells [64], also present on epithelium. Various minor adhesins, such as NadA (Neisseria adhesin A) also contribute to epithelial attachment and these have been described in detail elsewhere [60].

Having reached the potentially hostile environment of the nasopharynx, the meningococcus utilises several strategies to ensure persistence. As demonstrated in experimental systems, these are firstly, infection of epithelial cells by the pathogen and intracellular survival, which necessitates restoration of the encapsulated phenotype [65]. Secondly, the meningococcus also persists in the nasopharynx via encasement in biofilm [66]. In this setting, extracellular deoxyribonucleic acid (DNA) represents a major structural component of meningococcal biofilms, promoting binding of surface proteins and other extracellular polymers to form effective multicellular biofilms [66].

#### Translocation of the meningococcus to the lower airways

Notwithstanding dissemination to the lungs via the bloodstream or by person-to-person inhalation of contaminated aerosol droplets, microaspiration of biofilm-encased meningococci resident in the nasopharynx appears to represent the most probable mechanism of translocation of the meningococcus to the lower airways [2]. In this latter context, it is noteworthy that recent influenza virus infection in particular, as well as preceding pharyngitis, have both been linked to development of meningococcal pneumonia [67–69].

Given the seemingly lesser virulence of the meningococcus relative to that of exotoxin-producing bacterial respiratory pathogens such as *Streptococcus pneumoniae, Staphylococcus aureus* and *Pseudomonas aeruginosa*, the meningococcus may require a trigger to achieve full pathogenicity. In this context, the influenza virus is a particularly effective partner with respect to facilitating development of meningococcal pneumonia. This has been convincingly demonstrated in a murine model of experimental infection in which initial infection with influenza A virus was found to predispose for subsequent development of meningococcal bacteraemia [70]. Mechanisms by which preceding influenza virus infection may trigger development of meningococcal pneumonia include the following: i) inhibition of the mucociliary escalator [71]; ii) in the case of strains of the meningococcus belonging to sialic acid-containing capsular polysaccharide groups, viral neuraminidase mediates cleavage of capsular sialic acid, exposing underlying bacterial adhesins, facilitating adhesion to respiratory epithelium [61]; and iii) viral infection-associated elevated levels of pulmonary interferon-γ [70], which induce downregulation of expression of the alveolar macrophage class A scavenger, MARCO (macrophage receptor with collagenous structure) [72], an opsonin, which avidly binds unopsonised *N. meningitidis* via interaction with unidentified protein ligands [73].

Once established in the lower airways, the meningococcus utilises an array of virulence factors, which enable the pathogen to suppress or divert pulmonary host defences, resulting in establishment of severe infection.

#### Virulence mechanisms of the meningococcus

Notwithstanding the polysaccharide capsule and the IgA1 protease, the meningococcus possesses a range of additional virulence factors, which enable survival and proliferation of the pathogen in the lower airways. These include:

- Factor H-binding protein (fHbp), a 27-kDa surface-exposed lipoprotein, long recognised as being a key virulence factor of the meningococcus, is expressed by the majority of virulent strains of the pathogen [74]. With respect to its role in bacterial virulence, fHbp binds the major regulator of the alternative pathway of complement activation, fH, to the bacterial surface, thereby conferring protection against complement-mediated opsonophagocytosis and bacterial killing [74]. In addition, Porin A, one of two meningococcal porins, which play crucial roles in ion exchange, has also been reported to attenuate activation of the classical complement pathway via interaction with C4b-binding protein [75];
- Lipooligosaccharide endotoxin (LOS) is also a key virulence factor of the meningococcus. It is located in the outer membrane of the meningococcus and consists of a hydrophobic lipid A component linked to an outer hydrophilic oligosaccharide. As with other bacterial endotoxins, LOS possesses potent pro-inflammatory activities mediated primarily via interaction with the Toll-like receptor 4/MD2 complex on cells of the innate immune system, resulting in initiation of the MyD88-dependent signalling cascade and resultant production of a range of inflammatory cytokines/chemokines [76, 77]. Although potentially protective, the intensity of the LOS-activated inflammatory response is likely to result in lung damage, as well as dysfunction of pulmonary adaptive immune mechanisms. In addition, release of LOS contained in extracellular vesicles [78] may divert meningococcus-targeted immune mechanisms, while exacerbating harmful inflammatory responses. Moreover, binding of spontaneously released meningococcal outer membrane vesicles has been reported to prevent ensnarement of intact organisms by neutrophil extracellular traps in vitro [79];
- Modification of capsular size is also a strategy used by the meningococcus to evade adaptive immune mechanisms. In this context, it is noteworthy that the capsular polysaccharides of serogroups A, B, C, W and Y have been reported to interfere with activation of the classical complement pathway by preventing deposition of C4b following activation of complement by anti-capsular polysaccharide IgG and IgM antibodies, as well as via antagonism of binding of IgM antibodies targeted against LOS [80]. In addition, the weak immunogenicity of the capsular polysaccharide of serogroup B meningococcus also contributes to the virulence of this serogroup of the pathogen.

**Fig. 1:**
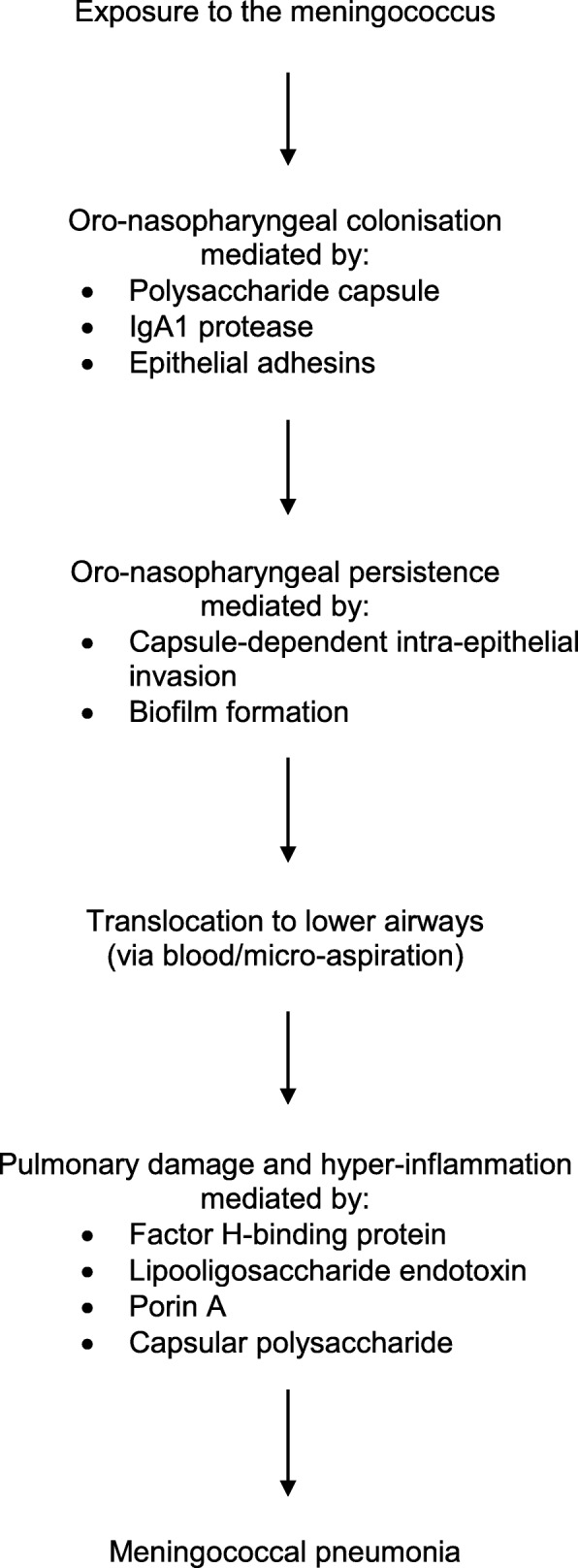
Possible stages in the pathogenesis of meningococcal pneumonia

The events involved in oro-nasopharyngeal colonisation and invasion of the lower airways by the meningococcus are summarised in Fig. 1.

### Clinical presentation

While meningococcal meningitis is the most common manifestation of meningococcal disease, primary pneumonia occurs in approximately 5–10% of patients with meningococcal disease [6, 7]. The clinical presentation of meningococcal pneumonia is indistinguishable from pneumonia caused by other infectious pathogens and while symptoms such as fever, chills and pleuritic chest pain are common, occurring in more than 50% of cases, productive cough and shortness of breath are less common [2]. A rash may occur in patients with pneumonia and associated sepsis, but meningococcaemia is a rare accompaniment of pneumonia [2]. Neither laboratory findings nor radiological features allow differentiation from other causes of pneumonia [2].

#### Case series - IMD

A number of case series has been reported over the years, describing either meningococcal disease, of which some patients had pneumonia [15, 81, 82], or specifically meningococcal pneumonia [19, 83]. Stephens and colleagues undertook a prospective, population-based surveillance of adult patients in Atlanta, US between 1 December 1988 and 30 November 1993 during which *N. meningitidis* was isolated from normally sterile sites [15]. Overall, 14/44 adults (32%) had pneumonia, sinusitis or tracheobronchitis as the likely source of bacteraemia, with pneumonia occurring in 10/44 (23%) cases. Seven of the 10 cases with pneumonia were > 50 years of age, while no pneumonias occurred in the 18–24 year old group. Serogroups Y, W-135 and B were the most common causes of pneumonia, as described elsewhere. Another surveillance study of IMD was undertaken in Dallas County, US, between 1992 and 1997 [81]. Overall, 151 patients were identified. Older patients (> 50 years) were more likely to have pneumonia (32% versus 4%; *p* = 0.0001) and less likely to have meningitis (p = 0.0001) when compared with younger patients. Among patients with pneumonia, there was a trend for more infections caused by serogroup Y. In addition, Hazarika and colleagues reported an outbreak of IMD in children in India between January 2008 and June 2009 [82]. Pneumonia occurred in 6.4% of cases.

#### Case series – meningococcal pneumonia

Putsch and colleagues studied hospitalised patients with pneumonia in Cleveland, US, during two time-periods in 1968, taking sputum or tracheal aspirates, which were cultured to optimise isolation of *N. meningitidis,* as well as acute and convalescent sera [83]. To determine carrier rates of the pathogen, throat cultures and sera were obtained from asymptomatic outpatients and sputum cultures from hospitalised patients without acute respiratory infection. The carrier rates were 7 and 13% in the initial and second periods, respectively. Overall, 47 patients, with a median age of 46 years, had acute pneumonia with the meningococcus isolated from sputum culture in 14 (30%) of cases. In addition, the meningococcus was isolated from sputum culture in seven of the 23 cases (30%) with respiratory infection other than pneumonia, but not from any of the patients without respiratory infection. The predominant serogroup in both patients with and without pneumonia was serogroup B. Winstaed and colleagues reviewed 58 cases of meningococcal pneumonia of all ages, 50 of which had previously been described in the literature, with eight new cases of their own added [19]. The median age was 57.5 years and 27 (52.9%) were males. Overall, 29 cases had prior underlying conditions (5 had other recent or concomitant infections, 4 patients had diabetes mellitus and 4 COPD, three patients were on corticosteroids [2 with asthma and 1 with systemic lupus erythematosus], 3 patients had myeloma or lymphoma and 2 cases had HIV infection). There were other, less common, associated medical conditions. Fever and chills and associated pleuritic chest pain occurred in > 50% cases, whereas productive cough and shortness of breath were less common. Various radiological findings were noted, including pleural effusion in six patients (13.3%). The most common serogroup isolated was Y (44%) followed by W135 (19.2%). A variety of antibiotic therapies was used. Complications included septic shock in two patients, while lung abscess, exudative pleural effusion, respiratory arrest, pericarditis and drug-induced neutropenia occurred in one patient each. The average age of the five (8.62%) patients who died was almost 20 years greater than that of the cases that survived.

#### Case reports – meningococcal pneumonia

A myriad of case reports of patients of all ages with meningococcal pneumonia has been published over the years from various regions of the world, including the US [4, 20, 42, 84–88], Europe [24, 89–97], the United Kingdom [98, 99], Russia [100], Japan [101–103], Taiwan [104], Australia [105], South America [106, 107] and Oman [69]. The characteristics of the patients with meningococcal pneumonia were similar to those described elsewhere in this review.

#### Other respiratory syndromes

Meningococcal infections have also been described as causing respiratory symptoms or conditions other than community-acquired pneumonia. One case study described a patient with meningococcaemia who presented initially as a non-specific “flu-like” syndrome, before significantly deteriorating [108]. In fact, presentation with upper respiratory tract symptoms, such as coryza and pharyngitis, together with fever and other symptoms, is a common early manifestation of meningitis, especially in children, and is followed subsequently by the classical features of meningitis or sepsis [6]. One study in a large district general hospital in the Netherlands reported 46 isolates of *N. meningitidis* cultured from the respiratory secretions of 44 patients, of whom one had pneumonia, 19 had acute respiratory infections and 18 had acute purulent exacerbations of chronic bronchitis [109]. The remaining cases, all of whom had purulent sputum, had a variety of symptoms. *N. meningitidis* has also been isolated in other studies from patients with acute and chronic bronchitis [110, 111], or other types of non-pneumonia lower respiratory tract infections [112]. Furthermore, *N. meningitidis* has also been documented as a cause of nosocomial pneumonia [113–116].

Among the respiratory complications that have been noted in patients with meningococcal pneumonia are cavitating lung infection [117], pleural effusion [118, 119], and empyema [120, 121], the latter case caused by a penicillin-resistant *N. meningitidis.*

### Laboratory diagnosis

Traditional microbiological culture techniques based on isolation of the meningococcus from saliva or sterile body fluids, usually blood or pleural fluid, remain the cornerstone of laboratory diagnosis of IMD [122]. It is very likely, however, that meningococcal pneumonia is underdiagnosed for several reasons: i) the pneumonia is often clinically indistinct from other pneumonias, such as pneumococcal pneumonias; ii) sputum is unreliable in diagnosis since it is difficult to differentiate asymptomatic oropharyngeal carriers from cases of pneumonia due to the meningococcus; and iii) blood cultures are relatively insensitive [2]. In the latter context, blood culture positivity rates in the setting of diagnosis of meningococcal pneumonia are variable, ranging from 6 to 79.3% [19, 31]. If validated and available, microbiological culture techniques may be supported by PCR-based approaches, including multiplex polymerase chain reaction (PCR) for simultaneous detection of meningococcal, pneumococcal and *Haemophilus influenzae* infection [122]. Latex agglutination tests for detection of meningococcal group-specific capsular polysaccharides in urine and cerebrospinal fluid, but not blood, are also available. However, these are of limited diagnostic application due to a high frequency of false-positive results, as well as failure to detect serogroup B meningococcal infection, which is common in many countries [122].

### Treatment of meningococcal pneumonia

Prior to 1991, penicillin was the treatment of choice for meningococcal infections [2]. However, with the emergence of penicillin-resistant strains in 1991 [123], in the setting of the high mortality associated with IMD, the empiric treatment recommendation was changed to a third generation cephalosporin [2]. Although not commonly done, should the microorganism be found to be sensitive to penicillin, therapy of IMD with high-dose penicillin may be considered. Concerningly, isolates of meningococci with decreased susceptibility to penicillin, as well as emerging resistance to other antibiotics, have been recognised in Spain, Europe, South Africa, the US, Canada and Brazil [123–131], with the latter study also reporting resistance of the meningococcus to ciprofloxacin. Alternative drug choices include meropenem (unavailability of a third generation cephalosporin), chloramphenicol (for severe beta-lactam allergy), aztreonam (if chloramphenicol is unavailable in the case of severe beta-lactam allergy), or a fluoroquinolone, such as moxifloxacin (currently restricted in the US, due to consideration of meningococcal fluoroquinolone resistance and lack of controlled trials in IMD) [7].

Although the optimal regimen for the treatment of meningococcal pneumonia *per se* has not been determined, it seems likely to be similar to that of IMD [2]. In fact, it has been noted that most cases of meningococcal pneumonia received penicillin before 1991 and that most received a cephalosporin after that date [19].

Corticosteroids have been used as adjunctive therapy in patients with meningitis, but their benefit appears to be evident in pneumococcal, rather than meningococcal, meningitis. Accordingly, these agents are not usually recommended in the clinical setting of meningococcal disease [2, 132]. Although there has been emerging evidence for the benefit of adjunctive corticosteroid therapy in patients with severe community-acquired pneumonia, there are no reports on the possible benefits of corticosteroids in severe meningococcal pneumonia [2].

### Prevention

#### Chemoprophylaxis

Chemoprophylaxis to eliminate nasopharyngeal carriage is recommended in close contacts of cases with meningococcal infection, in whom the risk of acquiring the infection is considerably elevated (400 to 800-fold higher than in the general population) [17]. Antibiotics that have been used include rifampicin, ceftriaxone, azithromycin and fluoroquinolones, although concerns about antibiotic resistance surround the use of rifampicin and the fluoroquinolones [5, 7, 17]. As indicated elsewhere, nosocomial cases of meningococcal pneumonia have been described, some of which appear to have developed following contact with patients who have meningococcal pneumonia [114, 115]. While the recommendations for prophylaxis are based on studies of contact with cases of meningitis, the epidemiology of meningococcal infection suggests that similar benefits may also be seen with chemoprophylaxis in the setting of contact with meningococcal pneumonia cases [8].

#### Immunization

Prevention of severe meningococcal disease is largely based on the immunoprophylaxis of meningococcal meningitis. As with prevention of invasive pneumococcal disease, two types of meningococcal capsular-based polysaccharides vaccines exist. Firstly, vaccines which consist solely of capsular polysaccharides derived from four of the major disease-causing serogroups of the meningococcus, specifically A, C, Y and W-135, and, secondly, conjugate vaccines in which the capsular polysaccharides are chemically linked to a suitable, immunogenic protein carrier, usually diphtheria toxoid, CRM197 [133]. According to the World Health Organization (WHO), meningococcal polysaccharide vaccines are bivalent (serogroups A and C), trivalent (serogroups A, C and W-135), or tetravalent (A, C, Y, W-135) [133]. As with pneumococcal polysaccharide vaccines, however, meningococcal purified capsular polysaccharide vaccines are also weakly immunogenic, being ineffective in the very young and failing to induce herd protection. Indeed, Sanofi-Pasteur recently announced discontinuation of production Menomune®, which is a tetravalent polysaccharide vaccine, approved in 1981 by the Food and Drug Administration (FDA) of the US for prevention of invasive meningococcal disease (serogroups A, C, Y and W-135) in high-risk individuals aged 2 years and older.

Currently two main types of meningococcal tetravalent conjugate vaccine exist. These are Menactra® and Menveo® licensed in the US in 2005 and 2010 respectively. Both contain purified meningococcal polysaccharides (A, C, Y and W-135) conjugated to CRM197. A similar vaccine, Nimenrix®, containing the same capsular polysaccharides conjugated to tetanus toxoid as protein carrier, is available outside the US. Relative to those vaccines containing purified polysaccharides only, the conjugate vaccines have improved immunogenicity, are suitable for use in very young children, prevent carriage and induce herd protection [133]. However, lack of coverage against serotype B remains a limitation of these vaccines.

To address this issue, two novel, capsular polysaccharide-free, protein-based vaccines have been developed, which specifically target serogroup B *N. meningitidis.* These are Trumenba® (MenB-FHbp) licensed for use in the US in 2014 and Bexsero® (4CMenB) licensed in the US, Canada, Europe and Australia, countries and regions in which invasive disease caused by *N. meningitidis* serogroup B remains a significant problem. Trumenba®, manufactured by Wyeth Pharmaceuticals Inc., is primarily recommended for immunisation of at-risk individuals aged ≥10–25 years and consists of the two recombinant variants of the meningococcal surface protein, fHBp [134, 135]. Bexsero®, manufactured by GlaxoSmithKline plc, is a more complex vaccine, which is comprised of three recombinant meningococcal serogroup B proteins combined with outer membrane vesicles derived from group B strain NZ98/254 [136]. Of the constituent proteins, two of these viz. fHbp and Neisseria heparin-binding protein (NHBA), are recombinant fusion proteins, while the third is the intact recombinant protein adhesin, NadA. The vesicles derived from strain NZ98/254 are enriched with a fourth immunogenic protein, PorA [136]. As with Trumenba®, Bexsero® is also recommended to protect those “at risk” individuals aged ≥10–25 years and has demonstrated protective efficacy ranging from around 64–78% in adolescents, as well as infants, having been introduced into the national immunisation programmes of the United Kingdom and Republic of Ireland [137–140].

With respect to novel potential vaccine candidate antigens, the IgA1 protease of the meningococcus, which shares a limited number of common epitopes with that of its pneumococcal counterpart, has shown promise in providing cross-protection against both bacterial pathogens in pre-clinical evaluation [141].

Table 2 describes the types and applications of meningococcal vaccines.

**Table 2:** Types and applications of meningococcal vaccines

Type	Vaccine antigen	Application	Refs
Purified capsular polysaccharide-based vaccines (not available for serogroup B)	i) Serogroups A,C (bivalent) ii) Serogroups A,C, W-135 (trivalent) iii) Serogroups A,C,Y,W-135 (tetravalent)	Weakly immunogenic; recommended for high-risk persons aged ≥2 years	[133]
Tetravalent capsular polysaccharide conjugate vaccines. Two types available: Menactra® and Menveo®	Serogroups A,C,Y,W-135 covered by both vaccines	Improved immunogenicity; suitable for immunization of young children; prevent nasopharyngeal carriage; induce herd protection	[134]
Protein-based, capsular polysaccharide-free vaccines targeting serogroup B. Two types available: Trumenba® and Bexsero®	i) Two recombinant variants of serogroup B fHbp (Trumenba®) ii) Three recombinant serogroup B proteins (fHbp; NHBA; NadA)* and vesicle-packaged Porin A (Bexero®)	High-risk persons aged ≥10–25 years High-risk persons aged ≥10–25 years and young children	[134–140]

*fHbp = factor H-binding protein; NHBA = Neisseria heparin-binding protein; NadA = Neisseria adhesin A

### Mortality

Meningococcal pneumonia appears to have a higher case-fatality rate than meningococcal meningitis (16% and 9–14%, respectively), with age (highest in patients > 65 years of age), specific serogroups (higher among serogroup W than serogroup B strains), and underlying disease, being important risk factors, much like other lower respiratory tract infections [2].

## Conclusions

The current review highlights the risk factors, pathogenesis, clinical features, diagnosis, treatment and prevention of meningococcal pneumonia. While many of these aspects have not been as well characterised as they have been for cases with meningococcal meningitis/invasive meningococcal infection, in general, they appear to be similar. Primary meningococcal pneumonia occurs in approximately 5–10% of patients with meningococcal disease and has identical clinical features to that of pneumonia caused by the other, more common pneumonia pathogens, including the pneumococcus. However, the diagnosis is most likely underestimated as a positive sputum is unreliable as it cannot differentiate asymptomatic colonisation from invasive infection, while blood cultures have varying sensitivity. The mortality of meningococcal pneumonia is higher than that for meningitis, with age (highest in the elderly), specific serogroups (higher among serogroup W than serogroup B strains), and underlying disease, being important risk factors. Current meningococcal vaccines do not provide coverage against all of the major disease-causing serogroups. In this context, innovation is needed with respect to development of vaccines, which provide serogroup-independent coverage.

## Data Availability

Data in the manuscript is derived from a literature review.

## Abbreviations

CD147: Extracellular matrix metalloproteinase inducer (also abbreviated to EMMPRIN)
CEACAM: Carcinoembryonic antigen cell adhesion molecule
COPD: Chronic obstructive pulmonary disease
CRM197: Non-toxic mutant of diphtheria toxin used as a carrier protein for polysaccharide vaccines
DNA: Deoxyribonucleic acid
FDA: Food and Drug Administration
FHbp: Factor H-binding protein
HIV: Human immunodeficiency virus
IgA1: Immunoglobulin A1
IMD: Invasive meningococcal disease
LOS: Lipooligosaccharide endotoxin
MARCO: Macrophage receptor with collagenous structure
MBL: Mannose-binding lectin
NadA: Neisseria adhesin A
NHBA: Neisseria heparin-binding protein
PCR: Polymerase chain reaction
US: United States of America
WHO: World Health Organization
